# Direct administration of 2-Hydroxypropyl-Beta-Cyclodextrin into guinea pig cochleae: Effects on physiological and histological measurements

**DOI:** 10.1371/journal.pone.0175236

**Published:** 2017-04-06

**Authors:** J. T. Lichtenhan, K. Hirose, C. A. Buchman, R. K. Duncan, A. N. Salt

**Affiliations:** 1 Washington University School of Medicine Department of Otolaryngology Saint Louis, Missouri, United States of America; 2 University of Michigan Kresge Hearing Research Institute Department of Otolaryngology-Head and Neck Surgery Ann Arbor, Michigan, United States of America; University of California Irvine, UNITED STATES

## Abstract

2-Hydroxypropyl-Beta-Cyclodextrin (HPβCD) can be used to treat Niemann-Pick type C disease, Alzheimer’s disease, and atherosclerosis. But, a consequence is that HPβCD can cause hearing loss. HPβCD was recently found to be toxic to outer hair cells (OHCs) in the organ of Corti. Previous studies on the chronic effects of *in vivo* HPβCD toxicity did not know the intra-cochlear concentration of HPβCD and attributed variable effects on OHCs to indirect drug delivery to the cochlea. We studied the acute effects of known HPβCD concentrations administered directly into intact guinea pig cochleae. Our novel approach injected solutions through pipette sealed into scala tympani in the cochlear apex. Solutions were driven along the length of the cochlear spiral toward the cochlear aqueduct in the base. This method ensured that therapeutic levels were achieved throughout the cochlea, including those regions tuned to mid to low frequencies and code speech vowels and background noise. A wide variety of measurements were made. Results were compared to measurements from ears treated with the HPβCD analog methyl-β-cyclodextrin (MβCD), salicylate that is well known to attenuate the gain of the cochlear amplifier, and injection of artificial perilymph alone (controls). Histological data showed that OHCs appeared normal after treatment with a low dose of HPβCD, and physiological data was consistent with attenuation of cochlear amplifier gain and disruption of non-linearity associated with transferring acoustic sound into neural excitation, an origin of distortion products that are commonly used to objectively assess hearing and hearing loss. A high dose of HPβCD caused sporadic OHC losses and markedly affected all physiologic measurements. MβCD caused virulent destruction of OHCs and physiologic responses. Toxicity of HPβCD to OHC along the cochlear length is variable even when a known intra-cochlear concentration is administered, at least for the duration of our acute studies.

## Introduction

2-Hydroxypropyl-Beta-Cyclodextrin (HPβCD) is a commonly used excipient to stabilize and solubilize pharmaceuticals. HPβCD reduces cholesterol and lipid accumulation, and has emerged as a possible treatment for Niemann-Pick type C disease, Alzheimer’s disease, and atherosclerosis [1–4]. But, a negative side effect of HPβCD-based treatments is hearing loss. Recent studies on the chronic effects of HPβCD administered systemically or directly into cerebrospinal fluid found graduated losses of outer hair cells (OHC) along the cochlear spiral, with more severe losses at the cochlear base than the apex [5–6]. While the origins of HPβCD toxicity within the ear remain uncertain, its effects can modulate cochlear or OHC electromechanics in excised cochleae [7–8]. In experiments reported here we used a variety of physiological measurements to understand the effects of acute administration of HPβCD, and HPβCD analog methyl-β-cyclodextrin (MβCD), directly into cochlear perilymph. Results were compared to measurements made during treatment with salicylate, which has effects that have been well characterized, and the effects of injecting artificial perilymph alone.

## Methods

### Animal preparation

We used NIH-strain pigmented guinea pigs of either sex (between 400–600 g). Initially animals were anesthetized with an intraperitoneal injection of sodium thiobutabarbital (100 mg/kg). Cutting shears were used to shave head and neck fur. A tracheotomy was performed, and the animal was artificially ventilated with isofluorane (~1% in oxygen) with respiratory volume maintained (5% end-tidal CO_2_). We monitored heart rate, O_2_ saturation, and expired CO_2_ level with a pulseoximeter/CO_2_ analyzer. The right cochlea was accessed with a ventral surgical approach. Soft tissue of the right ear canal was removed. The animal was mounted with hollow ear bars that allowed delivery of acoustic stimuli. A cannula placed in the left jugular vein was used to administer pancuronium bromide (0.06 mg/kg) to eliminate middle-ear muscle contractions just before the start of making auditory measurements. Body temperature was maintained (38°C) with a dc-powered heating blanket and rectal thermometer system. Experimental protocols for this study were approved by the Animal Studies Committee of Washington University (protocol numbers 20120113 and 20130069).

### Solution administration by injection into the cochlear apex

Ototoxic solutions were administered from a pipette sealed into the cochlear apex. Previously we have shown that apical injection drives solutions toward the cochlear aqueduct at the base of scala tympani, allowing the entire scala to be uniformly treated [9–11]. This overcomes the limitations of classic administration techniques to the cochlear base that do not reach therapeutic levels at the regions tuned to mid to low frequencies that code speech vowels and background noise. Administering into the cochlear base is certainly not the ideal approach to understand how HPβCD affects the entire cochlear length [11–16]. Solutions injected into the cochlear apex can be administered slowly if the goal of the experiment is to sequentially affect finely spaced cochlear regions contributing to a response [10–11]. For the experiments reported here, we used a constant, relatively fast (0.5 μL/min) injection rate.

Animals received either artificial perilymph alone (controls, n = 3 guinea pigs) or, in artificial perilymph, 20 mM salicylate (n = 3 guinea pigs), 13 mM HPβCD (n = 3 guinea pigs), 27 mM HPβCD (n = 3 guinea pigs), or 13 mM MβCD (n = 3 guinea pigs). The composition (in mM) of artificial perilymph was NaCl (127.5), KCl (3.5), NaHCO_3_ (25), CaCl_2_ (1.3), MgCl_2_ (1.2), NaH2PO_4_ (0.75), and Glucose (11) [10]. Animals treated with 13 mM HPβCD were in the “low-dose” group and those treated with 27 mM HPβCD were in the “high-dose” group. Salicylate alters surface cisternal system of hair cell bodies [17], reduces turgor pressure [18], and effects OHC motility [19]. In short, salicylate attenuates the gain of the cochlear amplifier. Solutions were injected from a pipette sealed into the cochlear apex in the 4^th^ cochlear turn. The mucosa covering the apex was removed with a damp, cotton wrapped applicator or cellulose wipe. Cyanoacrylate glue was applied to the dry bone of the apex, then a thin layer two-part silicone elastomer was applied. This effectively made the surface hydrophobic. The cochlear apex was fenestrated through the adhesive layers on the bone. The fenestration was made with a 30º, 1/3mm oval window pick by resting the pick on the surface at one location and then lifting the pick off the surface. The fenestra diameter was made to fit a 20–30 μm diameter tip pipette that was pulled from 100 mm x 0.58 mm inner-diameter glass tubing. We made a fluid-tight seal between the glass injection pipette and the hydrophobic surface by wicking fluid (accumulated from either condensation or cochlear fluid accumulation) and applying additional cyanoacrylate glue. Solutions were driven at 500 nL/min for 15 min for a total of 7500 nL through a 50 μL Hamilton gas-tight syringe (1710TLL), glued to a World Precision Instruments plexiglass coupler (MPH6S10), mounted on a computer-controlled World Precision Instruments Ultrapump.

### Acoustic stimuli and physiologic measurements

Electrophysiologic measurements were made using procedures that we have previously described [9–11]. Measurements were made with Tucker-Davis System 3 hardware controlled by custom-written software in Visual Basic (Microsoft) on a personal computer. TD-RP2 modules were used for stimulus generation. Stimuli were passed through TD-PA5 attenuators, and TD-HB7 headphone amplifiers. Acoustic stimuli were delivered in a closed sound system: an Etymotic ER-10C coupled to the hollow ear bar. Calibrations were completed in individual ears by tracking 70 dB SPL tones from 125 Hz to 26 kHz in ¼ octave steps. Cochlear response measurements were made differentially between an Ag/AgCl electrode in the round window niche and a platinum-needle electrode in the vertex. Measurements were made with an optically-coupled TD-HB7amplifier (1000X gain, 0.005–15 kHz bandpass filter), routed to TD-RP2 modules for digitization (48.8 kHz) and averaging. Animals were electrically grounded with an Ag/AgCl pellet electrode coupled to the exposed soft tissue of the neck by a fluid bridge.

### Histological preparation

At the conclusion of the apical injection experiments, the experimental cochleae were prepared for fixative injection. The pipette used to inject ototoxins was removed, and the fenestration was occasionally enlarged to a small extent to accept a 20–30 μm diameter tip pipette pulled from 100 mm x 0.58 mm inner-diameter glass tubing used to inject fixative. The pipette for fixative was sealed into the cochlea by wicking away any cochlear or condensation fluid accumulation while applying cyanoacrylate glue to form a fluid-tight junction. The round window membrane was perforated before the start of fixative injection. We injected 2.5% glutaraldehyde and 1.5% paraformaldehyde in a 0.065 M phosphate buffer. This solution was injected at 2000 nL / min for a total of 7500 nL. Cochleae were extracted and placed in fixative solution for at least two days at 4°C. Cochleae were decalcified (0.1 M EDTA with 0.4% glutaradehyde) for 14 days, osmicated (1% OsO_4_ in dH_2_O) for 60 minutes, dehydrated in ethanols and propylene oxide, embedded in Araldite resins, and sectioned parallel to the spiral axis of the fourth cochlear turn at 40 μM with a carbide steel knife. Sections were mounted in Permount on microscope slides and cover-slipped. Sections were analyzed with light microscopy by an author who was blinded to the treatment each ear received.

## Results

**Fig 1:**
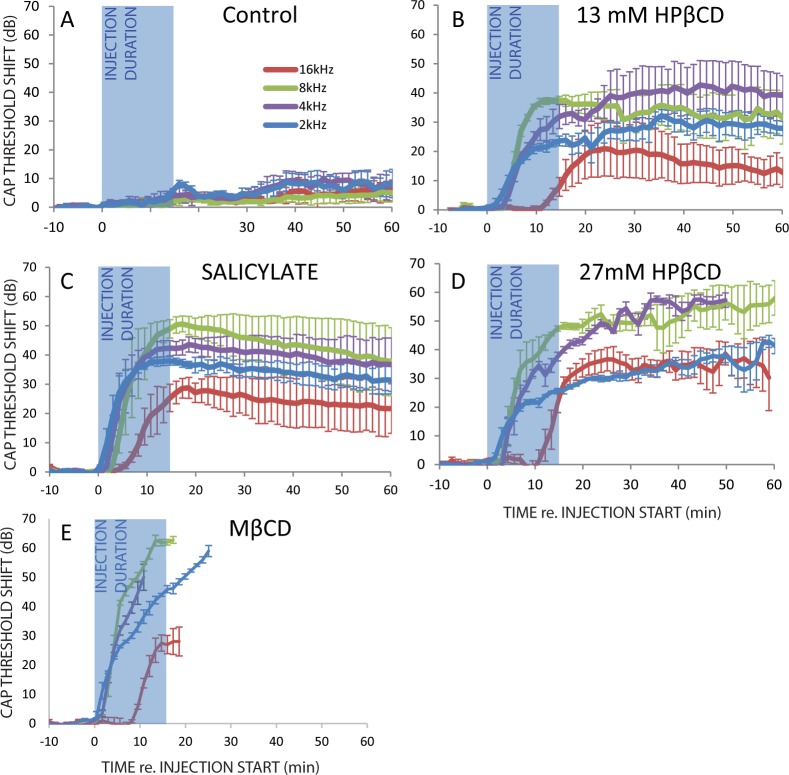
Cochlear action potential (CAP) threshold shifts as a function of time before, during, and after apical injection of various ototoxic solutions. Semi-transparent blue rectangles spanning 0 to 15 minutes identify when apical injections occurred. There were a total of three ears from three guinea pigs in each of the five groups.

The time course of cochlear action potential (CAP) threshold shifts differed across treatments and tone-burst frequencies (Fig 1). The ≤10 dB threshold shifts occurring 30 minutes after the start of injecting artificial perilymph alone (control experiments) quantify the extent to which the apical injection procedure itself influenced neural thresholds. The direction and rate (dB / min) of CAP threshold shifts differed between HPβCD 13 and 27 mM treatments, in that threshold shifts essentially raised and then plateaued for 13 mM treatment or, in contrast, steadily increased to a maximum or to the abolition of CAPs for 27 mM treatment. Quick CAP threshold shifts followed by gradual CAP recovery is consistent with the well-known temporary effects of salicylate (e.g., [20–21]. Maximal effects of salicylate and 27 mM HPβCD on CAP thresholds are consistent with elimination of cochlear amplifier gain, as targeted deletion of prestin in mice raise neural threshold by 40 to 60 dB [22–23]. Average threshold shifts of 20 to 40 dB from 13 mM HPβCD are far greater than the 6 dB threshold shifts caused by heterozygote prestin knockout mice when electromotility is halved [22]. Total CAP abolition during, or soon after, MβCD treatment is itself an indication of far greater effects than simple cochlear amplifier gain attenuation (see the histological data from these ears presented later). In normal ears CAP to low-level tone bursts originate from the peak of the traveling wave (reviewed in [24]). Across all toxic treatments, CAP thresholds to 2, 4, and 8 kHz started to rise soon after the injection start (i.e., zero minutes re. injection start), but ~3–8 additional minutes was needed to start abolishing CAP thresholds to 16 kHz. This demonstrates that we did not simultaneously treat the length of the cochlea. Comparing maximal effects on CAP threshold shifts in Fig 1 helps to understand the extent to which cochlear amplifier gain was attenuated across treatments.

**Fig 2:**
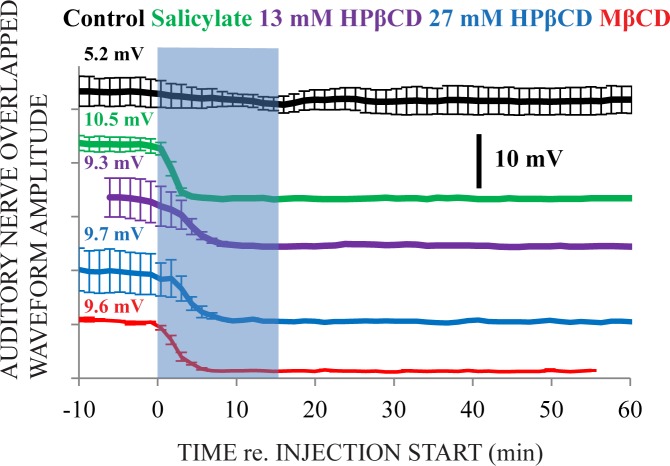
Auditory Nerve Overlapped Waveform (ANOW) amplitude measurements. The blue, semi-transparent rectangle identifies the 15 minute duration of apical injections. The values presented on the far left of the figure are average amplitude measurements from the ~10 minutes of data before the injection start. The ranking of measurements on the y-axis corresponds to the effect of the solution, with the measurements associated with the smallest effect at the top of the figure, progressing to the largest effect at the bottom of the figure. Salicylate, HPβCD, and MβCD treatments caused total abolition of ANOW, but artificial perilymph alone (control) did not. N = 3 for each treatment group.

Auditory Nerve Overlapped Waveform (ANOW) measurements (Fig 2) show that the apical injection technique can treat the apical half of the cochlear spiral, a region that classic round window administration cannot [11]. The ANOW originates from afferent auditory nerve fibers in the apical cochlear half and can quantify low-frequency auditory thresholds [10, 25]. ANOW amplitudes from supra-threshold, 50 dB SPL sound levels were ablated by salicylate, HPβCD, and MβCD treatments, but not by artificial perilymph alone (control; Fig 2). Subtle changes to ANOW from control ears during the time of injection suggest that perhaps some ANOW changes in ears treated with toxic solutions resulted from mechanical disturbances from the fast apical injection approach used for these experiments. Nevertheless, unlike CAPs, ANOW amplitude measurements were fully abolished with all four treatments, consistent with the ANOW being more sensitive than CAPs to cochlear manipulations and diseased states [26].

Cochlear microphonic (CM) measurements to 90 dB SPL 500 Hz tone bursts were made from inside the endolymphatic space where responses are not influenced by neural excitation to the sound used to evoke the desired hair-cell-based response [10]. Slight transient changes in CM amplitude from control, 13 mM HPβCD, and salicylate treatment are consistent with slight mechanical disruption caused by the relatively fast injection rate that was used here. The CM remaining after treatment with 27 mM HPβCD originates from current flow through remaining OHC and IHC, perhaps IHCs more than OHCs (as suggested from histological data below). Total abolition of CM measurements occurred for MβCD.

**Fig 3:**
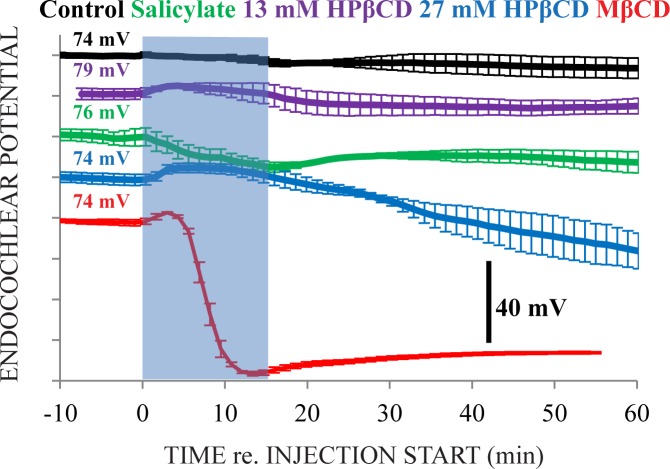
Endocochlear potential (EP) measured from inside the endolymphatic space of the third cochlear turn. The blue rectangle identifies the 15-minute injection. Voltages to the left of the rectangle are the average EP from ~10 minutes before the injection start. Measurement ranks along the y-axis indicate which solution caused the largest effect, smallest effects are at the top of the figure and largest effects at the bottom. Injection of artificial perilymph alone (control) did not affect EP. Injection of 13 mM HPβCD and salicylate caused slight and temporary effects. Treatment with 27 mM HPβCD did not abolish the EP but MβCD caused total abolition. N = 3 for each treatment group.

**Fig 4:**
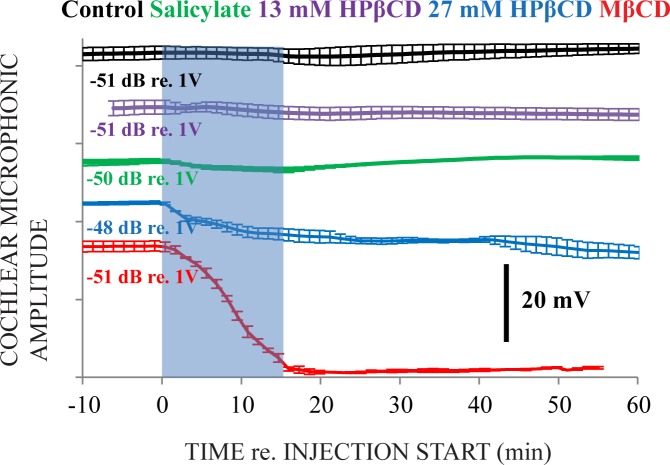
Cochlear microphonic (CM) measured from inside the endolymphatic space of the third cochlear turn. The semi-transparent blue rectangle identifies the 15 minute injection duration. Values listed on the far left of the figure are average amplitude measurements from the ~10 minutes of data collected before the start of injection. The ranking of measurements on the y-axis corresponds to the solution effect, with the measurements associated with the smallest effect at the top of the figure, progressing to the largest effect at the bottom of the figure. The noise floor for these CM measurements is approximately -85 dB, or ~35 dB down from the average amplitude measurements calculated from the 10 minutes of data before the injection start, which are those values presented on the far left of the figure. Artificial perilymph alone (control) and 13 mM HPβCD did not cause marked effects on CM amplitude, salicylate caused slight and temporary effects, 27 mM HPβCD did not fully abolish the CM but MβCD caused total abolition. Data were obtained from three ears for each treatment group.

Endocochlear potential (EP) measurements were made in the third cochlear turn (Fig 3). Artificial perilymph injection (control) did not markedly affect the EP. Treatment with 13 mM HPβCD and salicylate caused transient and temporary effects: 13 mM HPβCD caused EP to increase during injection and then returned to pre-injection values during the time immediately after injection while, in contrast, salicylate caused EP to decrease before gradually returning toward pre-injection values. Treatment with 27 mM HPβCD moderately affected the EP, causing transient increases during injection that returned to pre-injection values during the time immediately after injection before markedly declining further. MβCD caused a brief increase in EP then total abolition before the injection was complete. EP changes are mirrored by changes in the magnitude of the silent current, e.g., an EP decrease is consistent with a decrease in the standing current through outer hair cells in silence [27]. The time courses of effects on EP during treatment are not identical to the time courses of effects on CM (cf. Figs 3 and 4), which is consistent with the origin of these measurements being different even though they were made with the same electrode in the endolymphatic space of the third cochlear turn.

**Fig 5:**
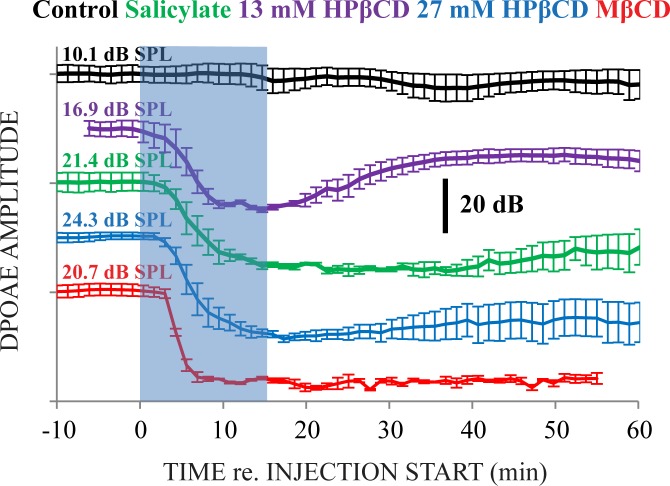
Distortion product otoacoustic emission (DPOAE) amplitudes at 2*f*_1_- *f*_2_ evoked from *f*_2_ ≈ 6 kHz, *f*_2_ / *f*_1_ = 1.2 kHz, *L*_2_ = 50 dB, *L*_1_ = 10 dB re. *L*_2_. The blue rectangle identifies the 15 minute injection. Values to the left of the rectangle are average DPOAE amplitudes from ~10 minutes of pre-injection data. The microphone noise floor was approximately -20 dB SPL. Measurement rankings on the y-axis were determined by the effect of the ototoxic solution: measurements associated with the smallest effect are at the top of the figure, progressing to the largest effect at the bottom of the figure. MβCD totally abolished DPOAEs, while the remaining ototoxic solutions caused transient declines in amplitudes that did not return to pre-injection levels. N = 3 for each treatment.

Cubic DPOAE amplitudes were measured at 2*f*_1_- *f*_2_ with an arbitrarily chosen *f*_*2*_ ≈ 6 kHz (Fig 5). Individual-ear DPOAE amplitude fine structure was considered by choosing the *f*_*2*_ primary-tone frequencies closest to 6 kHz that produced a peak in the 2*f*_1_- *f*_2_ DPOAE amplitude. Injection of artificial perilymph did not affect DPOAE amplitudes in control ears. With 13 mM HPβCD treatment, DPOAE amplitudes dramatically declined during the injection but gradually recovered toward near pre-injection levels. With salicylate and 27 mM HPβCD treatments, effects on DPOAE amplitude were longer, and did not fully recover. MβCD was the only treatment that totally abolished DPOAE amplitudes.

**Fig 6:**
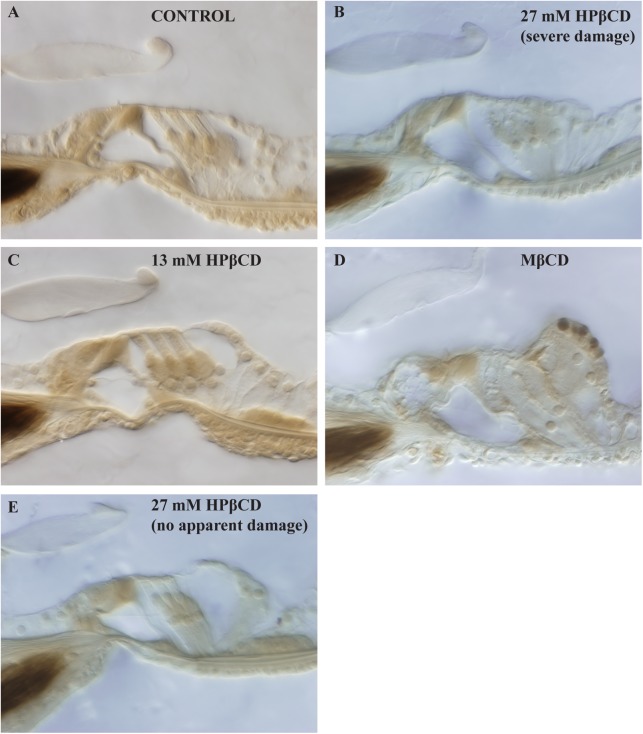
Histology of second cochlear turn organ of Corti from exemplar ears after apical injection of artificial perilymph (control, Panel A), 13 mM HPβCD (Panel B), 27 mM HPβCD (Panels C & D), and MβCD (Panel E). Histological assessment was not performed on ears receiving salicylate treatment. OHCs appeared normal in control ears and those receiving 13 mM HPβCD. Ears receiving 27 mM HPβCD had a variety to appearances, from normal to severe damage. All ears treated with MβCD had severe damage.

**Fig 7:**
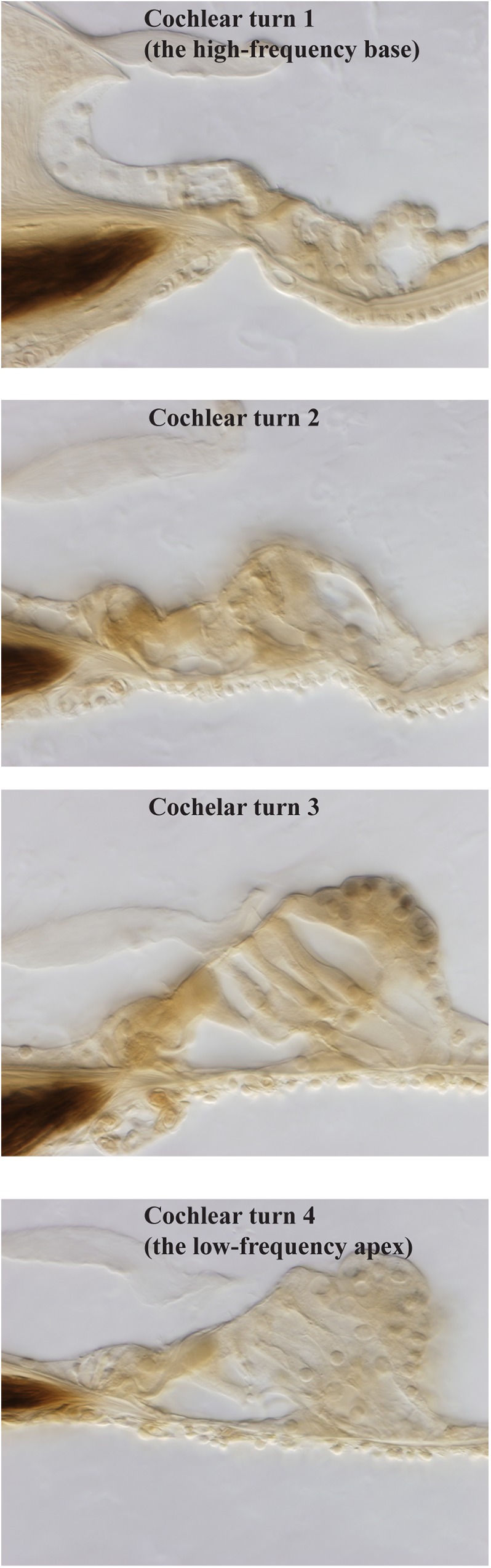
Organ of Corti histology from each of the four cochlear turns of one exemplar guinea pig ears after apical injection of 27 mM HPβCD. Damage was greater in the cochlear base that is tuned to high frequencies.

Sensory cells within the organ of Corti were evaluated using light microscopy and serial sections of plastic embedded cochleae (Fig 6). All sensory and non-sensory cells were well preserved in the control and 13 mM HPβCD groups. Effects from treatment with 27 mM HPβCD were variable, ranging from well-preserved to severely damaged outer and inner hair cells. Ears with severe IHC and OHC damage had greater effects at the base than apex (Fig 7). While some of this variability in OHC loss may be attributable to the relatively short time-frame of our acute study, interanimal variability is nevertheless consistent with that reported from Crumling et al. [5] and Cronin et al. [6] who studied the chronic effects of HPβCD treatment. Treatment with MβCD consistently caused severe damage directly to the OHCs and IHCs or the regions around these sensory cells. During the time course of these experiments with HPβCD, MβCD, or artificial perilymph (controls), lateral wall structures, including the spiral ligament fibrocytes, stria vascularis, spiral ganglion neurons did not appear to be altered. Specific morphometry was not performed in these structures, but there was no evidence of stria swelling or fluid leakage, the spiral ligament fibrocytes populated the ligament as in control ears, and there were no signs of myelin unwrapping from the spiral ganglion cell soma or evidence of cell swelling. Histological analysis was not performed for treatment with salicylate as the effects are well known or well-characterized [13, 21].

## Discussion

Our apical injection procedure overcomes the limitations of classical approaches of administrating drug treatments to the more surgically accessible cochlear base. In particular, apically injected solutions can treat the entire length of the cochlear spiral. We studied the acute effects of injecting HPβCD, and HPβCD analog MβCD, directly into the cochlea. We injected ~2x the volume of guinea pig scala tympani, most of which would have been driven out with our technique through cochlear aqueduct during the injection duration. The experiments ended and the animals were sacrificed 60 minutes after the start of treatment. Results were compared to those from salicylate treatments, as well as artificial perilymph alone (controls). Treatment with 13 and 27 mM HPβCD and salicylate ablated the ANOW amplitude and raised CAP thresholds by approximately 40 to 60 dB, consistent with attenuation of cochlear amplifier gain. Other measurements such as the endocochlear potential, cochlear microphonic recorded from inside the third cochlear turn endolymphatic space, distortion product otoacoustic emissions from the ear canal, and histological measurements generally showed that effects from MβCD were greatest, followed in order of decreasing effect by 27 mM HPβCD, 13 mM HPβCD, salicylate, and artificial perilymph injections alone (controls).

### HPβCD effects on an analysis of the electrical cochlear response

Here we address how treatment with 13 mM HPβCD affected harmonic distortion in the electrical cochlear response measured with a round window electrode. Sigmoidal, saturating, nonlinear functions are commonly used in analyses of empirical, gross measurements to study general transfer of acoustic sound into neural excitation (*f*_TR_, e.g., [9–10, 15, 24, 28–29]). Even and odd order distortions respectively associate with the asymmetry and saturation of *in vivo f*_TR_ [30] and analyses of distortions can be used to estimate the operating point of *f*_TR_ that is associated with the amplitude of distortion products (e.g., [31–32]. Expressing an even order distortion product as a function of operating point is proportional to the second derivative, and an odd order distortion product is proportional to the third derivative, of a function used to describe the sigmoidal, saturating, nonlinear *f*_TR_ (e.g., [30, 33–36].

**Fig 8:**
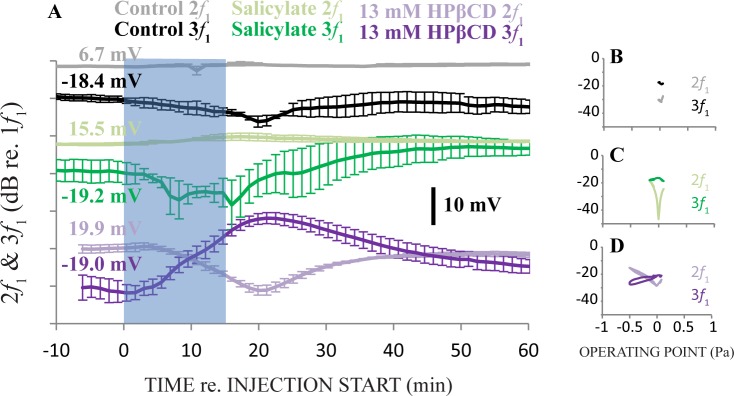
Panel A: Cochlear response harmonics recorded with a round window electrode to 90 dB SPL 500 Hz tone bursts. The semi-transparent blue rectangle identifies the 15 minute duration of solution injections into the cochlear apex. The ranking of measurements on the y-axis was determined by the effect of the solution on the amplitude of 3*f*_1_ of the cochlear response, with the measurements associated with the smallest effect at the top of the figure progressing to the largest effect at the bottom of the figure. N = 3 for each treatment. Panels B-D: Even and odd order harmonic amplitude measurements expressed as a function of operating point estimates nonlinearities involved with transferring acoustic sound into neural excitation (*f*_TR_). Even and odd order distortions from exemplar ears in the control and salicylate groups follow trajectories of the second and third derivative of a function that can describe *f*_TR_ and are consistent with previous experiments that injected gel into the cochlear apex to cause sustained displacement of the organ of Corti (Panels B&C). Effects from 13 mM HPβCD are unprecedented and cannot be explained by sustained displacement of the organ of Corti or apparent OHC loss (Fig 6C and Fig 6B), but the marked change operating point estimates inform the interpretation of affects *f*_TR_ are the likely origin of dramatic changes to DPOAE amplitude measurements (Fig 5).

Cochlear response harmonics to 90 dB SPL 500 Hz tone bursts were measured from animals in the control, salicylate, and 13 mM HPβCD groups (Fig 8). These are the experimental groups where cochlear response was still present after solution administration. Artificial perilymph injections (control) did not affect even order harmonic distortion (*f*_TR_ asymmetry, Fig 8A, gray), but caused a small and transient decrease in odd order harmonic amplitude (*f*_TR_ saturation) at ~18 min. after the injection start (Fig 8A, black). Salicylate caused a slight and brief increase in even order harmonic distortion amplitude immediately after injection (Fig 8A, olive), and a decrease in odd order harmonic amplitude that recovered to levels greater than pre-injection levels. (i.e., and “overshoot”; Fig 8A, green). HPβCD caused opposing effects in even and odd order harmonic distortion. The amplitude of even order harmonics maximally decreased at ~21 min after the injection start. In contrast, the amplitude of odd order harmonics increased to a maximum at ~22 min. Both even and odd order harmonics recovered to near pre-injection levels. We suspect that recovery of even and odd order harmonics, as well as slight recovery of DPOAE amplitudes, may have originated from minimal damage that precedes cell death and causes temporary functional deficit. These effects and recoveries suggest that a function which can describe the sigmoidal, saturating, nonlinearities involved with transferring acoustic sound into neural excitation was morphing during our acute experiments.

We will now use a Boltzmann analysis of electrical cochlear response measurements from exemplar ears to estimate the operating point of *f*_TR_ and express harmonic distortions as a function of operating point estimates. The cochlear response is a gross measurement of the cochlear microphonic from inner and outer hair cells, summating potentials, changes to the lateral wall potential from slow or sustained current through hair cells, excitatory postsynaptic potentials, onset or phase-locked compound action potentials from cochlear regions tuned to frequencies of the sound stimulus or those located more toward the base, and the coalescence with spontaneous excitation of single-auditory-nerve-fibers associated with cochlear regions that are not excited by the sound stimulus. We are thus studying cochlear nonlinearity in the general terms of influence on DPOAE amplitude, certainly not mechanoelectric transduction at the apical pole of an OHC as is commonly done with Boltzmann analyses. The Boltzmann analysis of the electrical cochlear response measurements provide a more unique perspective that builds on our qualitative description immediately above. Operating point estimates were obtained by adjusting Boltzmann parameters until the modeled output matched empirical cochlear responses. The Boltzmann function was V_t_ = V_EP_ + (− V_sat_ + 2 V_sat_ / (1 + exp(-2 S_B_ / V_sat_ (P_t_ + OP)))) where V_EP_ was a DC potential representing the endocochlear potential magnitude (mV), V_sat_ as the saturation voltage of the Boltzmann function (mV), S_B_ represented the slope of the Boltzmann function at its mid-point (mV/Pa), P_t_ represented the input pressure (Pa) as a function of time, OP represented the operating point of the Boltzmann function (Pa).

Harmonic distortions did not markedly vary in control ears when expressed as a function of operating point estimates (Fig 8B). Even and odd harmonic distortion variations with operating point estimates during salicylate injections (Fig 8C) were consistent with results from previous experiments that utilized gel injections into the cochlear apex to cause sustained displacement of the organ of Corti and simulate the effects of endolymphatic hydrops [33]. Variations to harmonic distortions expressed as a function of operating point estimates during 13 mM HPβCD injections were novel in that the trends deviated dramatically from the second and third derivative of the *in vivo f*_TR_ (Fig 8D). HPβCD caused unprecedented effects to physiologic measurements independent of apparent OHC loss (Fig 6B) that are not consistent with sustained displacement of the organ of Corti, such as what can happen if, for example, alteration to OHC bodies manipulate stereocilia coupling to the tectorial membrane [37].

**Fig 9:**
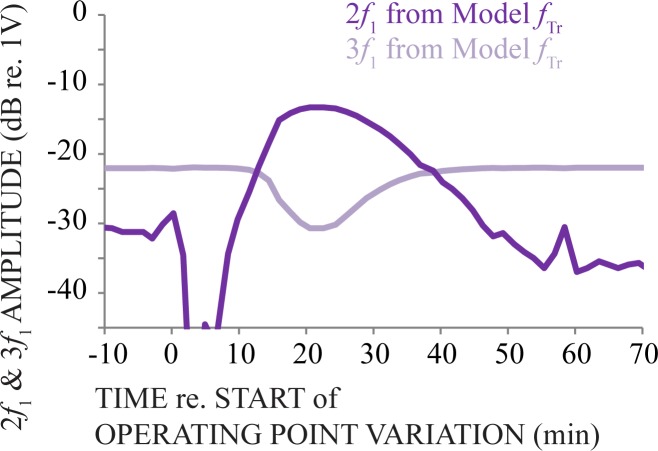
Simulated 2*f*_1_ and 3*f*_1_ harmonic distortion amplitude measurements from a Boltzmann analysis that used a sinewave input while OP was varied. OP estimates were obtained from a previous simulation (i.e., the x-axis values of Fig 8D). The trends of simulated 2*f*_1_ and 3*f*_1_ harmonics amplitudes expressed as a function of OP estimates are qualitatively similar to empirical harmonic distortion amplitude measurements made during apical injection of 13 mM HPβCD (Fig 8A, shades of purple). This simulation suggests that *in vivo* OP variations accounted for essence of changes to empirical harmonics measurements from 13 mM HPβCD treatment. Together with the results from the previous Boltzmann analysis (Fig 8B–8D), these results show that changes to the asymmetry, saturation, and sensitivity of *in vivo f*_tr_−and origin of distortion–can explain effects of 13 mM HPβCD on DPOAE amplitudes (Fig 5, purple).

Above we used empirical measurements in a Boltzmann analysis to understand how 13 mM HPβCD treatment changed operating point estimates of *in vivo f*_TR_. We now use the Boltzmann analysis to understand how these operating point estimates can describe the trends in 2*f*_1_ and 3*f*_1_ harmonic distortion measurements we found during the apical injection procedure (Fig 9). Recall that even (e.g., 2*f*_1_) and odd (e.g., 3*f*_1_) order harmonics respectively quantify the asymmetry and saturation of *in vivo f*_TR_. Using a Boltzmann analysis, we used a sinewave input (P_t_) and systematically varied OP with estimates obtained from fitting empirical data (i.e., the x-axis values of Fig 8D). The amplitude of the harmonics in the simulated output (V_t_) were measured and expressed as a function of OP (Fig 9). Results show that varying the operating point of the Boltzmann analysis yielded simulated harmonic distortions that were qualitatively similar to empirical harmonics (cf. Figs 8 and 7A, shades of purple). These results add additional support for the interpretation that 13 mM HPβCD caused morphing in the sigmoidal, saturating, nonlinearities involved with transferring acoustic sound into neural excitation during our acute experiments, without causing apparent OHC loss (Fig 6B).

### HPβCD effects on electrical cochlear responses help to understand effects on DPOAEs

**Fig 10:**
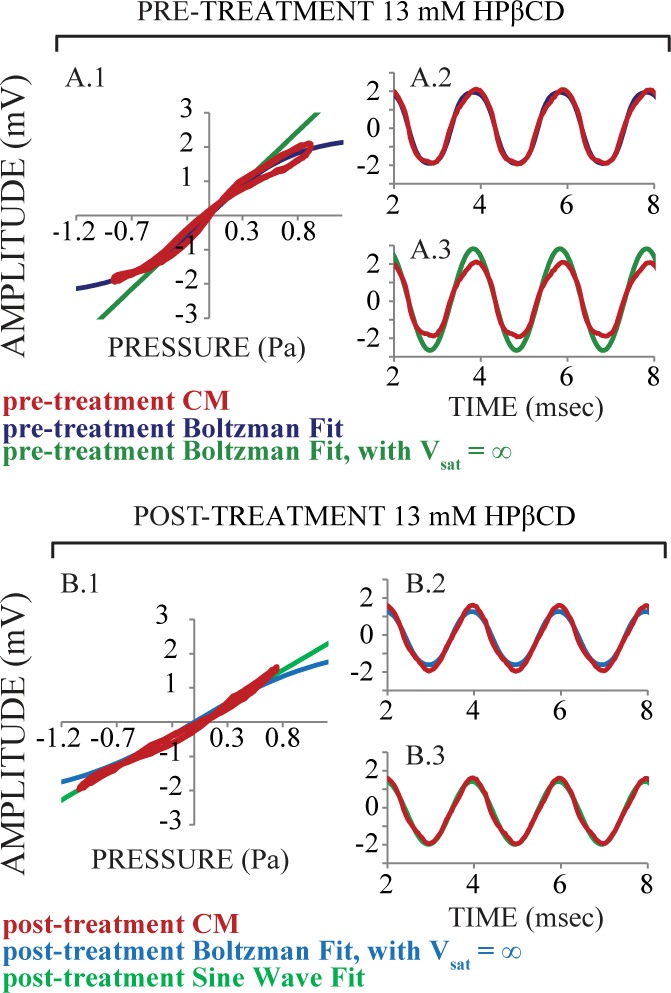
An analysis of cochlear microphonic (CM, red) measured from inside the endolymphatic space of the third cochlear turn in an exemplar ear before and after 13 mM HPβCD treatment. Pre-treatment (top panels): the best fit to the empirical CM was achieved with a typical Boltzmann analysis (Panel A, blue). Post-treatment (bottom panels): the best fit to empirical CM data was to a simple sine wave (Panel B, light green). These analyses suggest that mechanoelectric transduction was effectively linearized for the relatively high 90 dB SPLs used to evoke the CM. It is likely that saturation, or nonlinearity, of mechanoelectric transduction after 13 mM HPβCD treatment occurs at higher sound pressure levels that what we used here.

Here we discuss how the effects to the asymmetry, saturation, and sensitivity of *f*_TR_ related to the changes found to DPOAE amplitude measurements. Administering 13 mM HPβCD did not cause apparent changes to CM amplitude recorded inside the endolymphatic space but caused a large reduction of DPOAE amplitudes (cf. Figs 4 and 5 purple, ~15 minutes after the start of injection). These results suggest intact mechanoelectric transduction but marked attenuation of cochlear amplifier gain. We investigated this further by analyzing CM measurements made before, and 30 minutes after, the start of injection (Fig 10). Before treatment, the CM (Fig 10A, red) fit well to a typical Boltzmann function (Fig 10A, blue) but not to a Boltzmann function without saturation (V_sat_ from the Boltzmann analysis, Fig 10A, green). These fits to pre-treatment empirical CM can be seen both in the Boltzmann analysis (Fig 10A.1) and the time domain (Fig 10A.2 and 10A.3). During treatment, the CM (red) fit well to a simple sine wave (Fig 10B, light green). But, to achieve a fit to a Boltzmann function, V_sat_ had to be set to infinity (Fig 10B, light blue). These fits to empirical CM measurements made during treatment can be appreciated in both the Boltzmann analysis (Fig 10B.1) and the time domain (Fig 10B.2 and 10B.3). We suspect that saturation of CM amplitude would have occurred at sound pressure levels much higher than what was used for these experiments because there was no apparent loss of OHC stereocilia and bodies after 13 mM HPβCD treatment. The unexpected result of normal CM amplitude measured inside scala media in the face of maximal decreases in DPOAE amplitude during treatment of 13 mM HPβCD treatment likely originate from lack of saturation to the *in vivo f*_TR_. The near normal CM amplitude measured inside the endolymphatic space (Fig 4) and transient enhancement of EP measurements (Fig 3) made during 13 mM HPβCD treatment could be explained if decreased current flow through OHCs increases the overall resistance between endolymph and perilymph. That is to say, decreased current flow would reduce the OHC-generated potential, but with less shunting to perilymph the amplitude recorded from endolymph may not be reduced to the same degree.

### HPβCD does not affect the auditory nerve or lateral wall of scala media

Here we revisit the finding that ~40 min after the start of 13 mM HPβCD treatment DPOAEs returned to near-baseline amplitudes but CAP thresholds shifted to an asymptotic level that is consistent with fully attenuating cochlear amplifier gain (cf. Figs 1B and 4 purple). These findings naturally lead to the question: Does HPβCD cause an auditory neuropathy?

**Fig 11:**
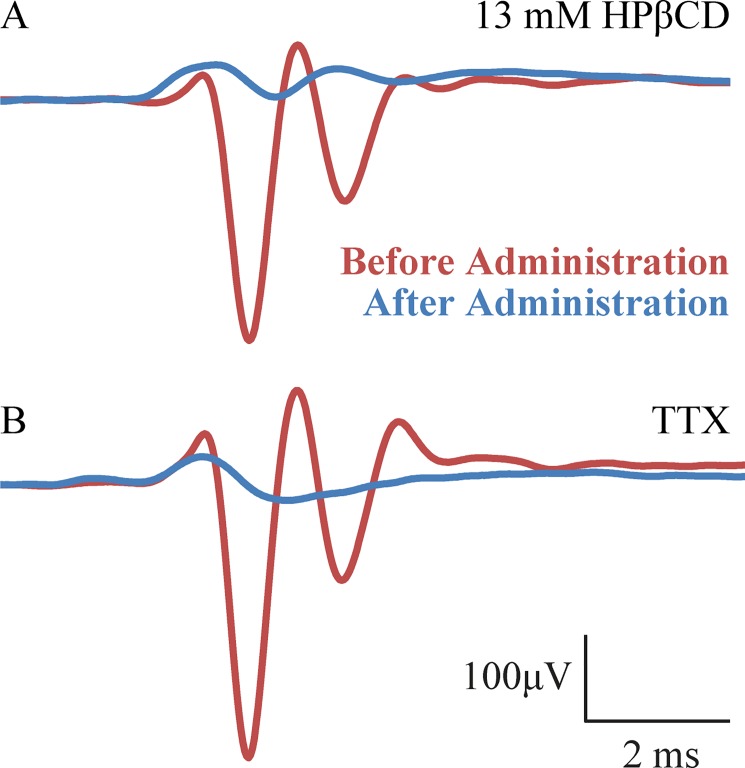
Cochlear response measurements from two different animals made before (red) and after (blue) treatment with HPβCD (Panel A) and TTX (Panel B) to 80 dB SPL 4 kHz tone bursts. Cochlear response waveform maintained CAP-like morphology after HPβCD treatment, consistent with reduced mechanical drive to neural excitation (Panel B, blue). In contrast, response waveform is EPSP-like following TTX treatment. Unlike TTX, results from HPβCD do not support the hypothesis that the auditory nerve is a site of action for 13 mM HPβCD.

Previous work found that intra-cochlear administration of the sodium channel blocker tetrodotoxin (TTX) can be used to study excitatory post-synaptic potentials (EPSPs) recorded with a round window electrode [38, 10]. EPSPs leading to normal spike generation have shorter latencies than CAP latencies. But, post-treatment latencies could decrease from broadened tuning caused by HPβCD affecting the cochlear amplifier, or from the need to increase stimulus level and thus probe a wider region of cochlear tuning curves to achieve a measureable response in damaged ears [39–40]. Broader filters have shorter delays than sharper filters [41]. TTX effectively causes an auditory neuropathy with raised neural thresholds in the face of normal DPOAE amplitudes and endocochlear potential [10]. It is thus possible that the voltages recorded during the asymptotic threshold shifts in Fig 1B were EPSPs masquerading as CAPs with an amplitude ≥ the typical 10 μV criteria for thresholds while DPOAEs amplitudes approximated pre-injection levels (cf. Figs 5 and 1B ~40 min after injection start). To address this possibility we compared round-window electrode measurements made before and after treatment with 13 mM HPβCD and 250 ng/ml TTX (Fig 11). We used measurements made within the first and last 10 minutes of a 70-minute experiment that used the same 15-minute injection procedure used in the other experiments discussed in this report. CAP measurements before HPβCD and TTX administration are comparable, as expected (Fig 11, red). After HPβCD treatment, the amplitude of the response was reduced but waveform morphology was more like the pre-HPβCD waveform than the post-TTX waveform, consistent with what would be expected by simple reduction of stimulus sound pressure level, or attenuation of cochlear amplifier gain, that drive neural responses (Fig 11A, blue). In contrast, after TTX administration the waveform resembles the well-known morphology of gross EPSPs as seen from a round-window ball electrode (Fig 11A, blue). *We conclude that HPβCD does not have its primary effect by directly acting on auditory neurons*.

CM amplitudes and endocochlear potential measurements made after the apical injection stopped were minimally affected by 13 mM HPβCD. The effects of HPβCD (and salicylate) on distortion products are thus different than those from furosemide which reduces both the DPOAE amplitudes *and* the EP [42]. *Our results do not suggest that HPβCD acts on the lateral wall of scala media*.

### Other theories on the origin of HPβCD ototoxicity

β-cyclodextrins can have widespread actions on cochlear function, potentially impacting any process involving cell stiffness or membrane biophysics. In other model systems, cyclodextrins can impact tight junctions [43], mechanotransduction [44] and synaptic function [45]. Without implicating any one of these, or potentially other interactions, it is clear that the effects of β-cyclodextrins can have multiple origins. While our data suggest the origins extend beyond outer hair cells, it remains unclear whether targets are permanently affected by β-cyclodextrins.

### Do HPβCD effects differ between species and along the length of the cochlear spiral?

Histological data from 27 mM HPβCD treatment showed that severe OHC and IHC damage was more pervasive in the basal cochlear half than the apical half (Fig 7), a finding that is consistent with that found by Crumling et al. [5] and Cronin et al. [6] who studied the chronic effects of HPβCD in mice by administering systemically and directly into cerebrospinal fluid. We suspect Cronin et al.’s drug entered the cochleae in cerebral spinal fluid through the cochlear aqueduct in the base of scala tympani, and was therefore at a lower concentration in the apical half of the cochlear length (cf. studies of chronic effects on measurements from mid-frequencies with administration to the cochlear base [13,21]). In contrast, it is unlikely that our solutions were diluted by mixing with cerebral spinal fluid because our 500 nL / min injection rate is larger than the ~30 nL / min sustained entry of cerebral spinal fluid through the cochlear aqueduct [46]. Methodological differences aside, our histological data showing severe hair cell damage to be more pervasive at the cochlear base than at the apex is consistent with Crumling et al. [5] and Cronin et al.’s [6] findings and suggests that *the graded effects of HPβCD on histological measurements along the cochlear length do not differ between species*.

Histological data alone cannot determine if HPβCD has varying effects on *measurements of hearing* along the cochlear length. CAP thresholds to the highest tone burst frequency (16 kHz) were affected less than to the lowest tone burst frequency (2 kHz) by 13 mM HPβCD (Fig 1B) and salicylate (Fig 1C), but were similarly affected by 27 mM HPβCD (Fig 1D). Our neural threshold measurements are not consistent with those from Crumling et al. [5] and Cronin et al. [6] who found that auditory brainstem response thresholds to low-frequency tone burst (4 kHz) were shifted by ~40 dB after HPβCD treatment but those to high-frequency tone burst (16 & 32 kHz) shifted ~50–60 dB. The difference between our results and those from Crumling et al. [5] and Cronin et al. [6] might be explained by their low-frequency baseline (control) thresholds that are slightly high in the strain used [47], a result attributed to greater OHC death. Or, disagreeing data may originate from differences in our methods used to study the acute effects of HPβCD and their methods used to study the chronic effects of HPβCD. Since our sets of histological data agree in that HPβCD has a lesser effect in the cochlear apex, one would ideally want to study the chronic HPβCD effects with an approach that could ensure treatment of the entire cochlear length in many different species and use physiologic measurements that can be obtained throughout the cochlear length. But, as it currently stands, the presently available physiological data suggests that *the graded effects of HPβCD on measurements of hearing from along the cochlear length do differ between species*.

Coupling together the available, the histological and physiological findings leads to the hypothesis that apical cells may be less susceptible to death, but are equally susceptible to functional deficit. This hypothesis predicts a continuum of a dose-response gradation from functional deficit to cell death. Addressing the possibility of longitudinal gradients in cell physiology may be a promising area of study, particularly once the perilymph concentrations of clinically applied HPβCD are known.

## Conclusions

We studied the acute effects of HPβCD injected directly into the perilymph of intact and sealed cochleae. We found that a low-dose of HPβCD raised CAP to an extent that was consistent with attenuating cochlear amplifier gain, had no apparent effect on the EP, altered general nonlinearities involved with transferring acoustic sound into neural excitation without causing apparent OHC loss, and the CM measured from inside the endolymphatic space. In contrast, DPOAEs measured in the ear canal were greatly diminished. A high-dose of HPβCD elevated CAP thresholds, markedly affected the EP, CM and DPOAEs, caused sporadic OHC losses that were consistent with previous studies on the chronic effects of HPβCD. Neither the low- or high-dose of HPβCD caused apparent disruption of the scala media lateral wall or the auditory nerve. But, for the duration of our acute studies, known intra-cochlear concentrations high-dose of HPβCD caused variable effects on OHCs throughout the length of the cochlear spiral.
